# Synthesis of inverse ringwoodite sheds light on the subduction history of Tibetan ophiolites

**DOI:** 10.1038/s41598-018-23790-9

**Published:** 2018-04-03

**Authors:** Luca Bindi, William L. Griffin, Wendy R. Panero, Ekaterina Sirotkina, Andrey Bobrov, Tetsuo Irifune

**Affiliations:** 1 Dipartimento di Scienze della Terra, Università di Firenze, Via La Pira 4, I-50121 Florence, Italy; 2 CNR-Istituto di Geoscienze e Greorisorse, Via La Pira 4, I-50121 Florence, Italy; 3 ARC Centre of Excellence for Core to Crust Fluid Systems and GEMOC, Department of Earth and Planetary Sciences, Macquarie University, Sydney, NSW 2109 Australia; 4 School of Earth Sciences, Ohio State University, Columbus, Ohio, USA; 5 Vernadsky Institute of Geochemistry and Analytical Chemistry of Russian Academy of Sciences, Moscow, 119991 Russia; 6 Department of Petrology, Geological Faculty, Moscow State University, Moscow, 119991 Russia; 7 Institute of Experimental Mineralogy of Russian Academy of Sciences, Chernogolovka, 142432 Russia; 8 Geodynamics Research Center, Ehime University, Matsuyama, 790–8577 Japan; 9 Earth-Life Science Institute, Tokyo Institute of Technology, Tokyo, 152–8550 Japan

## Abstract

Tibetan ophiolites are shallow mantle material and crustal slabs that were subducted as deep as the mantle transition zone, a conclusion supported by the discovery of high-pressure phases like inverse ringwoodite in these sequences. Ringwoodite, Mg_2_SiO_4_, exhibits the normal spinel structure, with Mg in the octahedral A site and Si in the tetrahedral B site. Through A and B site-disorder, the inverse spinel has four-coordinated A cations and the six-coordinated site hosts a mixture of A and B cations. This process affects the density and impedance contrasts across the boundaries in the transition zone and seismic-wave velocities in this portion of the Earth. We report the first synthesis at high pressure (20 GPa) and high temperature (1600 °C) of a Cr-bearing ringwoodite with a completely inverse-spinel structure. Chemical, structural, and computational analysis confirm the stability of inverse ringwoodite and add further constraints to the subduction history of the Luobusa peridotite of the Tibetan ophiolites.

## Introduction

The recent discovery of ringwoodite in chromitite bodies of the Luobusa peridotite in Tibet establishes, together with other evidence, that while the chromitites formed at shallow depths, they later were subducted to depths equivalent to the mantle transition zone (MTZ; 440–660 km)^1,2^. This ringwoodite, however, labeled “BWJ phase”, is dissimilar to ringwoodite as synthesized in the laboratory or observed in meteorites^3^: its structural and chemical characterization showed that this ringwoodite exhibits an inverse-spinel structure. Normal ringwoodite of the spinel structure has tetrahedrally coordinated Si, with octahedrally coordinated Mg. However, structural refinement of the “BWJ phase” showed the mineral to have the spinel structure with Si in the octahedral site and Mg disordered between the octahedral and tetrahedral sites^1^. Its composition, (Mg_0.70_Si_1.15_Fe_0.03_Al_0.02_)Mg_0.90_O_4_, also is noteworthy for the presence of minor but significant amounts of Fe and Al, which were suggested^1^ to be crucial for the stabilization of the inverse-spinel structure. The “BWJ phase” has a density of 3.3 g/cm^3^, lower than the classic ^A^Mg_2_^B^SiO_4_ ringwoodite (3.6 g/cm^3^) and approaching that of wadsleyite (around 3.4 g/cm^3^).

Mantle peridotites are mainly composed of olivine^4^ in the upper mantle, and pressure-induced transitions at depth transform olivine to wadsleyite (β-phase). This transition is responsible for the globally observed 410 km discontinuity. At higher pressures, wadsleyite transforms to ringwoodite (γ-phase). The transition from wadsleyite to spinel-structured ringwoodite occurs at approximately 520 km depth and finally, ringwoodite breaks down to ferropericlase ((Mg,Fe)O) plus bridgmanite (MgSiO_3_; perovskite-type structure) at an average depth of 660 km^5^. Among all these structural transitions, the wadsleyite to ringwoodite transformation is the most enigmatic as it does not produce any global seismic reflection. This is unexpected as the contrast in density and compression and shear velocities from wadsleyite to ringwoodite should be large enough to be observable^6^. However, the stabilization of an inverse-spinel structure as the product of the wadsleyite transformation could explain the absence of a visible seismic reflection within the MTZ.

Although natural ringwoodite is quite common in shocked meteorites^7^, the recent finding of a terrestrial ringwoodite included in a super-deep diamond^8^ has provided geological evidence of the existence of this phase in the deep Earth. Unfortunately, the size of the crystal did not allow the collection of high-quality structural data. Structural data collected on lab-grown ringwoodite or by first-principles computational studies show that the spinel-structured ringwoodite exists in a very dense topology based on a slightly distorted closed packed oxide anion lattice with Si cations hosted within the tetrahedral B site and Mg at the octahedral A sites^9^.

Here we report H*P*-H*T* synthesis experiments in the magnesiochromite (MChr)–forsterite (Fo) system together with first-principles calculations, and show that the experimentally-produced ringwoodite exhibits the same inverse-spinel structure as the BWJ natural phase. The structure of the synthetic inverse ringwoodite seems to be stabilized by the presence of chromium, which may play the same role as Fe and Al in the natural BWJ phase.

## Results

### Crystal-chemical considerations

**Figure 1:**
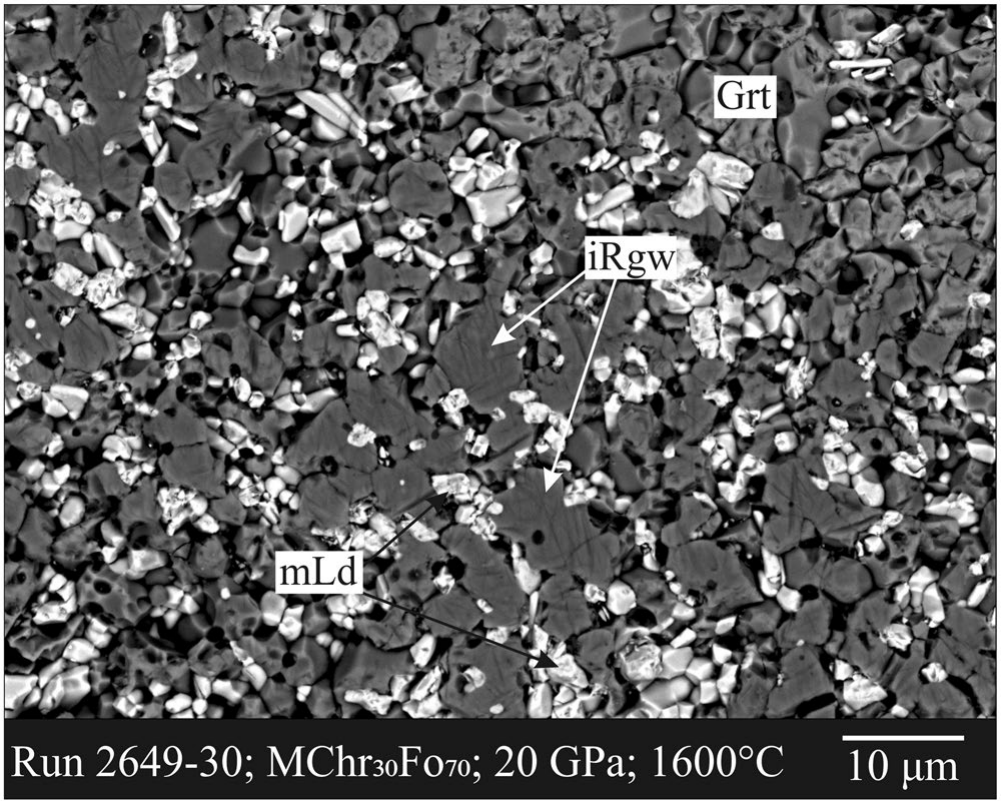
The studied experimental sample. SEM-BSE image of the experimental run (#2649–30) synthesized at *P* = 20 GPa and *T = *1600 °C with the magnesiochromite (MChr)–forsterite (Fo) composition MChr_30_Fo_70_. Inverse ringwoodite (iRgw) dominated the run products, accompanied by Mg_2_Cr_2_O_5_ (mLd) and garnet (Grt).

A small ringwoodite fragment (hereafter iRgw) was handpicked from the polished section of the experimental run 2649–30 (Fig. 1) under a reflected light microscope and mounted on a 5 μm diameter carbon fiber, which was, in turn, attached to a glass rod. Single-crystal X-ray diffraction reveals that the fragment has the structure of iRgw: topologically identical to those reported for normal γ-Mg_2_SiO_4_^9,10^ with a space group symmetry *Fd*-3*m*, with A-cations occupying the 16*d* position (point symmetry-3*m*), and the B-cations the 8*a* position (point symmetry -43*m*). Anions are hosted at the 32*e* position (point symmetry 3*m*). The cubic unit-cell volume is 558.74(4) Å, 1.4(1)% less than reported for the natural BWJ spinel^1^ (i.e., 566.8 Å), yet 5.9% greater than normal ringwoodite^10^ (525.7 Å). The Mg-tetrahedron shows a mean bond distance of 1.945(4) Å, slightly larger than the tetrahedral Mg in åkermanite^11^, Ca_2_MgSi_2_O_7_ (1.92 Å). The mixed (Mg,Si)-octahedron exhibits a mean bond distance of 1.970(2) Å, intermediate between the value of 2.072 Å observed for normal ringwoodite^10^ (with all Mg in the octahedral site) and 1.757 Å observed for stishovite^12^. Similar values were obtained for the B-O and A-O mean bond distances for the natural BWJ spinel^1^, i.e. 1.945 Å and 1.984 Å, respectively. All the Cr is concentrated in the octahedral site together with Mg and Si, consistent with the typical coordination with oxygen of Cr^3+^. Likewise, in the BWJ spinel^1^ the minor Fe and Al were found to be disordered at the octahedral site together with Mg and Si.

Both synthetic and natural (i.e., BWJ phase) inverse ringwoodite exhibit a *u* parameter (*x*-coordinate of the oxygen atom) greater than 0.25 (i.e., 0.2613 and 0.2607, respectively). As *u* increases above 0.25, the anions move away from the nearest tetrahedral cation in the [111] direction^9^, thereby increasing the size of the tetrahedron relative to the undistorted close-packed anion arrangement but without changing its overall -43*m* symmetry. At the same time the octahedra become smaller and assume -3*m* (rather than *m*3*m)* symmetry, and the lengths of the six edges shared with adjacent octahedra become shorter relative to the unshared edges (which remain essentially constant in length). This geometry reflects the cation disordering observed in inverse ringwoodite.

Given the significantly different sizes of Mg and Si (ionic radius of 0.72 and 0.40 Å, respectively^13^), and the fact that they coexist in the same octahedral site in inverse ringwoodite, one could expect an ordering of the two cations, lowering the symmetry from cubic to tetragonal. The *Fd*-3*m* → *P*4_1_22 transformation has been observed in several spinels, like qandilite^14^, Mg_2_TiO_4_, where the difference in ionic radii between Mg and Ti^4+^ is less than in inverse ringwoodite. Nevertheless, we did not observe any deviation of the unit-cell parameters from the cubic symmetry, and the refined oxygen thermal ellipsoid is not significantly anisotropic in both synthetic and natural iRgw, which is inconsistent with a high static positional disorder from mixing of Si and Mg on the octahedral site. 16*d* sites with Mg and Si are indeed more distorted^15^ (σ^2^ = 33.6 and 29.8, for synthetic and natural iRgw) than pure Mg sites^10^ (σ^2^ = 7.4), yet there is no net deviation for the overall structure.

### First-principles calculations

**Figure 2:**
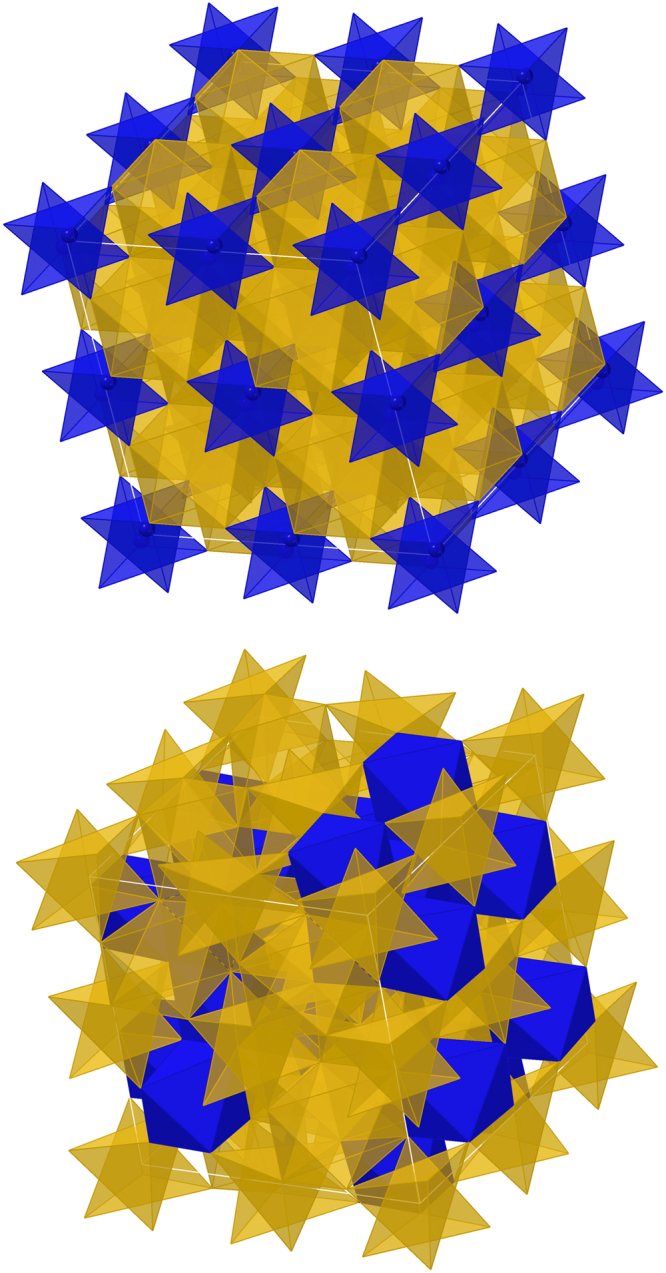
Crystal structure. (top) Normal ringwoodite with Si in tetrahedral sites (blue) and Mg in octahedral sites; (bottom) One of the randomly generated structures in which all tetrahedral sites are filled with Mg and half the octahedral sites are filled with Si.

At 20 GPa, the enthalpy of each of 10 randomly generated configurations (Fig. 2) are 83–128 kJ/mol greater than for normal ringwoodite at the same pressure, with an ensemble average excess enthalpy of 97.6 kJ/mol. A stabilizing factor of *TS*_config_ of 28.3 kJ/mol at 1600 °C results from the differences in configurational entropy between the fully ordered normal ringwoodite and the inverse phase with the distribution of Si on half the octahedral sites^6^. Therefore, the pure endmember ringwoodite is unlikely to form as inverse ringwoodite unless additional entropic contributions were to contribute. However, the incorporation of 6.25% Cr_2_O_3_, or 0.125 Cr per formula unit, into the same 10 randomly-generated structures reduces the ensemble average excess enthalpy to 58.4 kJ/mol. If the effect of Cr_2_O_3_ as a function of composition is linear, then 0.08 Cr per formula unit would have an excess enthalpy of about 72.5 kJ/mol. At the same time, the additional configurations of Cr increase the *TS*_config_ at 1600 °C to 36.8 kJ/mol (34.4 kJ/mol for 8%), nearly comparable to the excess enthalpy. The incorporation of Cr_2_O_3_ therefore stabilizes the inverse structure relative to the normal structure in two ways: (1) the radius of Cr^3+^ in VI-fold coordination is intermediate between those of Mg^2+^ and Si^4+^, yet greater than either in IV-fold coordination, and (2) the increase in configurational entropy resulting from a second compositional component. Taken together, these two factors nearly explain the stability of inverse ringwoodite with minor trivalent cations in the octahedral sites. Considering the possibility of Cr^3+^ defects substituting as non-nearest-octahedral site neighbors will likely increase the enthalpy of defect formation moderately (e.g. Panero *et al*.^16^) due to balancing defect charges over longer distances. Given the Cr_2_O_3_ concentrations considered here, the difference in configurational entropy is less than 2 kJ/mol and therefore unlikely to be a significant contributor to further stabilization.

**Figure 3:**
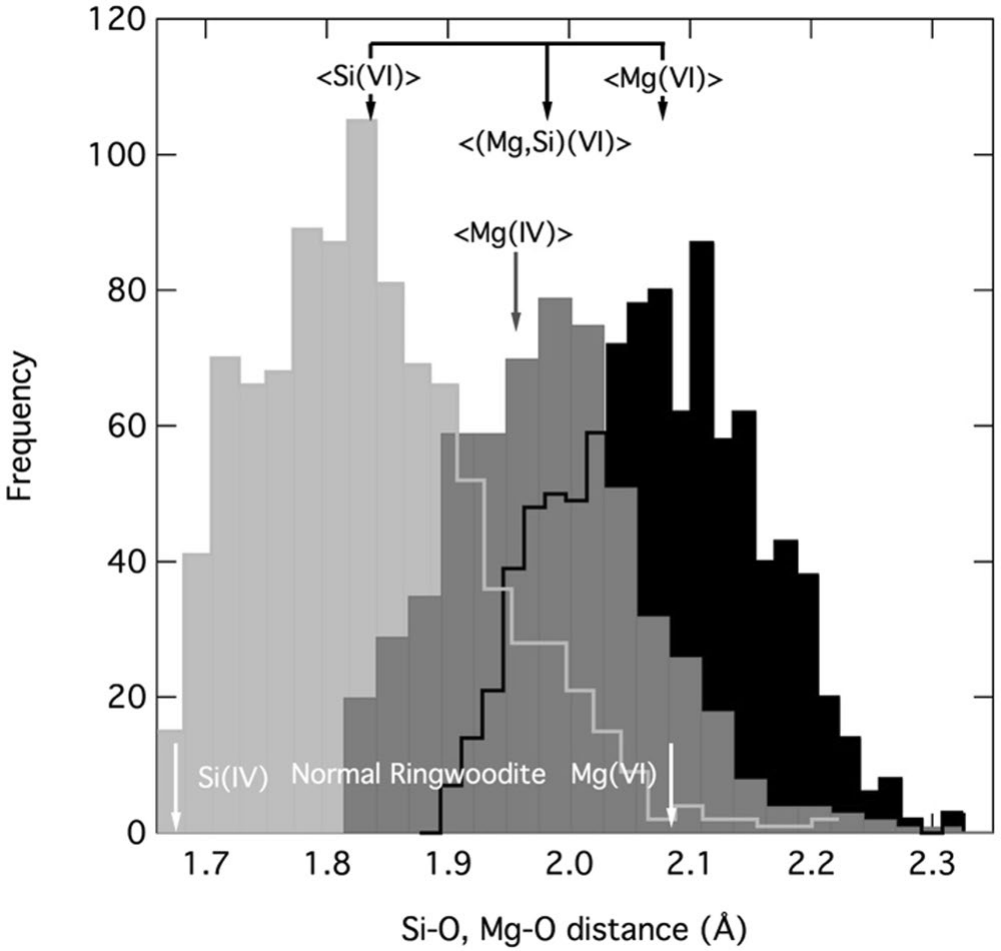
Bond distances in the crystal structure. Histogram of ^VI^Si-O (light grey), ^VI^Mg-O (black), and ^IV^Mg-O distances (dark grey) at zero pressure for each of the ten configurations calculated. Average values for each are marked, as well as the mean oxygen distance for all occupied octahedral sites. Corresponding distances for normal ringwoodite, relaxed using the same computational parameter, is shown in white along the bottom.

The ensemble average cubic unit-cell parameter of the inverse spinel is 8.1929 Å compared to the calculated normal ringwoodite unit-cell parameter of 8.1324 Å, reflecting a 2.2 vol% lattice expansion. Calculated tetrahedral and octahedral bond lengths in normal ringwoodite are similar to those measured: 1.675 Å and 2.084 Å, respectively. However, in the inverse case, the larger Mg cation expands the average bond length in the tetrahedral site to 1.9824 Å, while the ensemble average octahedral site decreases to 1.982 Å. The octahedral sites occupied by Si have an average bond length of 1.836 Å while those occupied by Mg have a longer bond length (2.0768 Å; Fig. 3).

### Discussion and implications for the Luobusa peridotite

The Luobusa massif is one of many peridotitic bodies along a 1000-km stretch of the Yarlong-Zhangbo suture in southern Tibet, formed during the massive continental collision that formed the Himalayas and the Tibetan Plateau. Luobusa has been the object of detailed mineralogical investigations for over 30 years, following the discovery of a remarkable assemblage of ultra-high-pressure (UH*P*) and ultra-reduced minerals included in the chromite ores (chromitite) of the massif^17^. While the peridotites and their associated chromitites have petrological features and geochemical signatures typical of low-pressure (a few km depth) generation in the mantle wedge above subduction zones, they also contain evidence of UHP metamorphism, followed by rapid exhumation to the surface.

Evidence for metamorphism in or near the upper part of the MTZ includes (1) exsolution of pyroxenes and rare coesite from chromite, suggesting inversion from a high-*P* polymorph of chromite; (2) microstructures suggesting that the present chromitites recrystallized under static conditions from fine-grained, highly deformed mixtures of wadsleyite and an octahedral polymorph of chromite^2^; (3) harzburgites with coarsely vermicular symplectites of opx + Cr–Al spinel ± cpx. Reconstructions suggest that these are the breakdown products of majoritic garnets, with estimated minimum pressures to >13 GPa^1^.

Numerous octahedral grains (50–200 µm) made up of clinochlore, lizardite^18^ or antigorite were separated from the Luobusa chromitites, and referred to as ‘pseudomorphs after cubic olivine’^19^. Most such grains are cloudy to opaque, but Griffin *et al*.^1^ re-examined transparent grains and recognized these as the phase termed ‘BWJ’, with the inverse-spinel structure, iRgw.

Given that the chemical composition of natural iRgw, with a small excess of Si and a deficit of Mg, as well as small amounts of Al, corresponds to that of an anhydrous antigorite, Griffin *et al*.^1^ suggested that it formed through the subduction-dehydration of antigorite, equilibrating under conditions broadly consistent with the formation of ringwoodite or wadsleyite.

However, because neither ideal ringwoodite nor wadsleyite structures exhibit Si in octahedral coordination, we suggest that iRgw may have instead originated from a higher-pressure phase with pre-existing SiO_6_ groups, and transformed at moderate temperature conditions, so that the Si-O bond reorganization is kinetically hindered. In this regard, a new post-spinel phase with composition Mg(Mg,Cr,Si)_2_O_4_ synthesized in the model system MgCr_2_O_4_–Mg_2_SiO_4_ at 16 GPa and 1600 °C has been recently reported^20^. The compound was found to crystallize with a distorted orthorhombic calcium-titanate (CaTi_2_O_4_) structure type, space group *Cmc*2_1_, and shows six-fold coordinated silicon. We suggest that both natural iRgw and synthetic post-spinel phase^20^ are representative of the podiform chromitites in the Luobusa ophiolite, which contain diamond and other former ultrahigh-pressure minerals^2,19,21,22^. Yamamoto *et al*.^23^ suggested a UH*P* precursor with a calcium ferrite structure that originally formed at a pressure of >12.5 GPa and then decomposed to low-pressure chromite containing silicate exsolutions. The stability range of this polymorph and its ability to incorporate large amounts of Ca and Si have been demonstrated experimentally^24^. In contrast, Ishii *et al*.^25^ suggested a lower-pressure origin for these chromitites because they did not contain the assemblage Mg_2_Cr_2_O_5_ + Cr_2_O_3_ (Fe_2_Cr_2_O_5_ + Cr_2_O_3_). The new Mg(Mg,Cr,Si)_2_O_4_ post-spinel phase^20^, which contains significant amounts of the Mg_2_SiO_4_ component never reported before in a post-spinel phase, may be an intermediate product in the deep recycling of silicate-bearing UH*P* chromitites^26^ and the source of the recovered iRgw, which might be formed during the inverse transformation from the new post-spinel phase Mg[(Cr, Mg)(Si, Mg)]O_4_ to chromite during mantle upwelling.

The present work, by confirming the stability of an inverse ringwoodite at conditions of the MTZ, adds further constraints to the subduction history of the Luobusa peridotite, and many other peridotites along the Yarlong-Zhangbo suture. It confirms that shallow-mantle material, as well as crustal slabs, can be subducted into the transition zone, heated to ambient temperatures, and then exhumed, probably by a combination of its own buoyancy and the strong upwelling that accompanies slab rollback^1^. The recognition of this process, and its verification by experimental studies, provides new evidence for the deep subduction and re-exhumation of lithospheric mantle. It identifies a major geodynamic process that may accompany large-scale plate collisions worldwide, and points out a potential indicator mineral for such processes in other peridotite bodies associated with such collisions.

## Methods

### Synthesis

High-pressure synthesis was performed at *P* = 20 GPa and *T* = 1600 °C using a 2000-t Kawai-type multianvil apparatus at the Geodynamics Research Center, Ehime University, Japan. The sample was compressed by eight cubic tungsten carbide anvils with 3-mm truncation edge lengths (TEL). The sample was heated in a tubular LaCrO_3_ heater. The sample was loaded into a platinum capsule isolated from the heater by a MgO insulator. The pressure medium was a semi-sintered (Mg, Co)O octahedron of 8.0 mm in edge length. The cell assembly used in the present experiment is described in detail by Sirotkina *et al*.^27^.

Temperature was measured using a W_97_Re_3_-W_75_Re_25_ thermocouple. Pressure was calibrated at room temperature based on semiconductor-metal transitions of Bi, ZnS and GaAs^28^. The effect of temperature on pressure was further corrected using the α-β and β-γ phase transitions of olivine^29,30^. The accuracy in determination of pressure and temperature is estimated to be ±0.5 GPa (30) and ±10 °C, respectively.

The starting material was prepared for the magnesiochromite (MChr)–forsterite (Fo) composition corresponding to the stoichiometry MChr_30_Fo_70_. Very fine powders (<100 nm) of MgO, SiO_2_, and Cr_2_O_3_ (purities 99.99%) were mechanically mixed using an agate mortar. The prepared mixture was dried at 100 °C for 24 hours and enclosed in a Pt capsule. Then, the sample was pressurized and heated in the multianvil apparatus. The run duration was 4.5 h, which is long enough to reach chemical equilibrium of coexisting phases according to our earlier studies^27,31^ using such ultrafine oxide starting materials. The sample was rapidly quenched to ambient temperature by switching off the power supply with a quench rate of 200 °C/s.

The recovered experimental product (#2469–30) was sliced into two pieces. One piece was embedded in epoxy, polished with diamond pastes, and then analyzed by scanning electron microscope and electron probe micro-analyzer. Another piece was subjected to XRD study. Inverse ringwoodite (iRgw) prevailed in the run product, accompanied by Mg_2_Cr_2_O_5_ (mLd) and garnet (Grt) (Fig. 1).

### Scanning electron microscopy

The instrument used was a Zeiss - EVO MA15 Scanning Electron Microscope coupled with an Oxford INCA250 energy-dispersive spectrometer, operating at 25 kV accelerating potential, 500 pA probe current, 2500 cps as average count rate on the whole spectrum, and a counting time of 500 s. Samples were sputter-coated with 30-nm-thick carbon film.

### Electron microprobe

A preliminary chemical analysis using energy dispersive spectrometry, performed on the same crystal fragment of inverse ringwoodite used for the structural study (see below) as well as on other fragments from the same run product, did not indicate the presence of elements (Z > 9) other than Cr, Mg and Si. The chemical composition was then determined using wavelength dispersive analysis (WDS) by means of a Jeol JXA-8600 electron microprobe. We used 40 s as counting time. The matrix correction was performed with the Bence-Albee^32^ program as modified by Albee and Ray^33^. The standards employed were forsterite (Mg, Si) and synthetic Cr_2_O_3_ (Cr). The crystal used for the X-ray study was found to be homogeneous within the analytical uncertainty. The average chemical composition (six analyses on different spots) is (wt %), SiO_2_ 55.02(19); MgO 40.18(18); Cr_2_O_3_ 4.23(11); total 99.43(22); corresponding, on the basis of 4 oxygen atoms, to [Mg_1.96(5)_Si_0.96(3)_Cr_0.08(3)_]O_4_.

### Single-crystal X-ray diffraction and structure refinement

Single-crystal X-ray studies were carried out using a Oxford Diffraction Xcalibur 3 diffractometer equipped with an Oxford Diffraction CCD detector, with graphite-monochromatized Mo*K*α radiation (λ = 0.71073 Å), working conditions 50 kV × 50 nA and with 200 s exposure time per frame; the detector-to-sample distance was 6 cm. Inverse ringwoodite is cubic, space group *Fd*-3*m*, with unit-cell parameters: *a* = 8.2364(2) Å, *V* = 558.74(4) Å^3^, and *Z* = 8.

Single-crystal X-ray diffraction intensity data were integrated and corrected for standard Lorentz and polarization factors with the *CrysAlis* RED package^34^. The program ABSPACK in *CrysAlis* RED^34^ was used for the absorption correction. A total of 268 unique reflections was collected. The structure was refined starting from the atomic coordinates reported for ringwoodite^10^ using the program Shelxl-97^35^. Given the observed larger unit-cell volume compared to ringwoodite^10^ (i.e., 527.3 Å^3^), the site occupancy factor (s.o.f.) at the cation sites was allowed to vary (Si *vs* structural vacancy for the A and B sites, respectively) using scattering curves for neutral atoms taken from the *International Tables for Crystallography*^36^. The use of scattering curves for ionized atoms (e.g., Mg^2+^, Fe^2+^, Si^2.5+^, Si^3.5+^, Si^4+^, O^1.5−^, O^2−^) did not change the refined electron numbers at the A and B structural sites. In terms of mean electron number, we obtained 13.4 and 12.0 *e*^-^ for the octahedral and tetrahedral site, respectively. For this reason, noting the close similarity between the scattering power of Si and Mg, we hypothesized that all the Mg is ordered at the tetrahedral B-site and that a site population of Mg_0.48_Si_0.48_Cr_0.04_ is accommodated at the A-octahedral site on the basis of the observed bond distances. Such a distribution is also in agreement with the observed overall geometry of the A and B sites and in accord with the fact that Cr^3+^ has a strong crystal-field-stabilization effect and prefers to enter the octahedral site. The site populations were then fixed accordingly in the subsequent cycles of the refinement. At the last refinement stage, with anisotropic atomic displacement parameters for all atoms and no constraints, the residual value settled at *R*_1_(*F*) = 0.0219 for 75 observed reflections [*F*_o_ > 4σ(*F*_o_)] and 7 parameters and at *R*_1_(*F*) = 0.0221 for all 87 independent reflections.

Crystallographic data (CCDC 1586933) can be obtained free of charge from *The Cambridge Crystallographic Data Centre* via www.ccdc.cam.ac.uk/data_request/cif.

### First-principles calculations

Calculations were performed using the projector-augmented wave method (PAW)^37^ of the density functional theory as implemented in the Vienna ab-initio Simulation Package (VASP) package^37,38^. We employed the generalized-gradient approximation (GGA) in the Perdew-Burke-Ernzerhof^39^ formulation for the exchange-correlation part, with a kinetic energy cutoff of 800 eV. Calculations at zero and 20 GPa were performed on a 2 × 2 × 2 super cell of the primitive lattice of each normal ringwoodite and inverse ringwoodite (112 atoms). Calculations containing Cr were performed using GGA + U with a *U*_eff_ = *U* − *J* of 3.5 eV^40^, representing the on-site Coulomb repulsion, *U*, and the electron-electron interaction, *J*. Inverse ringwoodite is modeled with all the tetrahedral sites occupied by magnesium, and the octahedral sites are then occupied by 50% magnesium and 50% silicon. The arrangement of the ^A^Mg- and ^B^Si-sites is distributed randomly, and a set of 10 configurations was calculated at each pressure following the approach of Panero and Caracas^41^.

Relative to normal ringwoodite, we calculate the partition function of inverse ringwoodite at 20 GPa,

1 $$Z=\sum _{i}{e}^{-\beta {E}_{i}}$$

where *E*_*i*_ is the energy of configuration *i*, and β is (*k*_*B*_*T*)^−1^. The probability of each state is therefore a function of both the energy of the state and the temperature,

2 $${P}_{i}=\frac{1}{Z}{e}^{-\beta {E}_{i}}$$

The ensemble average energy at that composition is then calculated directly from the partition function,

3 $$\langle E\rangle =\sum _{i}{E}_{i}{P}_{i}\,$$

and lattice constants weighted similarly according to the energy partition function. Ensemble average volume and bond lengths are calculated similarly.

We treat the configurational entropy as a counting of microstates in which each octahedral site is independent. We neglect the configuration on the vibrational component as evidenced by the similarity in the Raman spectra, addressing only the effects of configurational entropy. We then infer the Gibbs free energy of formation of inverse ringwoodite relative to that of normal ringwoodite at 20 GPa to be

4 $${G}_{inverse}=(\langle E\rangle -TS)-{G}_{normal}$$

where the ideal configurational entropy of the fully inverted structure is 15.1 J/mol/K^6^.

The increased stabilization of the structure through the incorporation of Cr is assessed through the substitution of 2 Cr^3+^ for neighboring Mg^2+^ and Si^4+^ cations for the normal and lowest-energy inverse spinel structures. For the supercell considered, a single substitution represents 6.25 mol% Cr_2_O_3_ in ringwoodite. While nearest-neighbor defects are considered here, non-locally balanced charges in the case of non-nearest neighbor Tschermak defects have an additional defect enthalpy of ~15% in ilmenite and bridgmanite^16^.
